# Immersive bilingualism reshapes the core of the brain

**DOI:** 10.1007/s00429-016-1307-9

**Published:** 2016-09-27

**Authors:** Christos Pliatsikas, Vincent DeLuca, Elisavet Moschopoulou, James Douglas Saddy

**Affiliations:** 1 Department of Clinical Language Sciences, School of Psychology and Clinical Language Sciences, University of Reading, Reading, RG6 6AL UK; 2 Department of Psychology, School of Psychology and Clinical Language Sciences, University of Reading, Reading, RG6 6AL UK

**Keywords:** Bilingualism, Basal ganglia, Thalamus, Structural MRI, Immersion

## Abstract

Bilingualism has been shown to affect the structure of the brain, including cortical regions related to language. Less is known about subcortical structures, such as the basal ganglia, which underlie speech monitoring and language selection, processes that are crucial for bilinguals, as well as other linguistic functions, such as grammatical and phonological acquisition and processing. Simultaneous bilinguals have demonstrated significant reshaping of the basal ganglia and the thalamus compared to monolinguals. However, it is not clear whether these effects are due to learning of the second language (L2) at a very young age or simply due to continuous usage of two languages. Here, we show that bilingualism-induced subcortical effects are directly related to the amount of continuous L2 usage, or L2 immersion. We found significant subcortical reshaping in non-simultaneous (or sequential) bilinguals with extensive immersion in a bilingual environment, closely mirroring the recent findings in simultaneous bilinguals. Importantly, some of these effects were positively correlated to the amount of L2 immersion. Conversely, sequential bilinguals with comparable proficiency and age of acquisition (AoA) but limited immersion did not show similar effects. Our results provide structural evidence to suggestions that L2 acquisition continuously occurs in an immersive environment, and is expressed as dynamic reshaping of the core of the brain. These findings propose that second language learning in the brain is a dynamic procedure which depends on active and continuous L2 usage.

## Introduction

Despite the increasing amount of recent evidence for the effects of bilingualism on the structure of the cortex, the cerebellum, and the white matter tracts (Pliatsikas et al. 2014a; Abutalebi et al. 2015; Olsen et al. 2015; Olulade et al. 2015; Mamiya et al. 2016), very few studies have reported any effects of bilingualism on the shape and/or volume of subcortical structures, such as the basal ganglia and the thalamus. Considering the role that has been attributed to subcortical structures for L2 learning, and especially phonological processing and language switching (Green and Abutalebi 2013; Abutalebi and Green 2016), the absence of structural effects might appear as a paradox, especially given the amount of evidence that has been provided for cortical areas which are also central to L2 learning.

Indeed, of the available Voxel-based Morphometry (VBM) studies to date, only two have reported increased volume of subcortical structures for bilingual compared with monolingual participants. Zou et al. (2012) compared bimodal bilinguals of Chinese spoken and sign language to monolingual speakers of Chinese. They reported increased volume in the head of the left caudate nucleus (LCN) for bilinguals, compared with monolinguals. In the same study, Zou and colleagues also reported increased activation of the LCN in bilinguals, in a task that required them to switch between producing sign and spoken language, compared with a non-switching task; in addition, the functional activation of the LCN was significantly correlated to its volume. This led the authors to suggest that the LCN is crucial for language switching in bimodal bilinguals, an effect already reported in unimodal bilinguals (Crinion 2006; Abutalebi et al. 2008), and that language switching incurs structural changes in the area. This interpretation is in accordance with the suggestion that the LCN is central in the selection among language alternatives in bilinguals (Green and Abutalebi 2013; Abutalebi and Green 2016). It is worth noting here that the volume of the head of the LCN has also been shown to positively correlate with phonemic fluency in L2 in bilinguals (Grogan et al. 2009). This finding further confirms the role of the LCN in language selection and switching, suggesting that increased LCN volume contributes to reduced interference from the native language (L1), which, in turn, enhances the performance in a phonological task in L2.

The only other VBM study that has presented significant subcortical between-groups differences is by Abutalebi et al. (2013). In that study, a group of female multilinguals demonstrated increased volume in the left putamen, compared with monolinguals. This effect was accompanied by increased activation for multilinguals of the left putamen in a picture naming task in their third language (L3) only, but not their L1 and L2. Moreover, the volume of the left putamen positively correlated to the multilinguals’ performance in their L3. The age of acquisition (AoA) of the three languages might be of particular importance: whereas these participants acquired their L1 and L2 (German and Italian) early in life (defined as “kindergarten age”), they acquired their L3 (English) formally at school and after the age of 10. These differences in AoA between the three languages might suggest that the left putamen is more engaged in the processing of languages that are learnt later in life, or those languages in which the bi-/multilinguals are less proficient (Abutalebi et al. 2013). Since the putamen has been linked to articulatory processing in bilinguals (Frenck-Mestre et al. 2005), the above results might signify structural and functional changes as a result of increased articulatory demands that are faced by late learners of a language.

It is clear that the available evidence for the structural effects of bilingualism on subcortical structures is rather scarce and not comparable to the available evidence for cortical regions. Burgaleta et al. (2016) suggested that this is because of the preferred method of use in the majority of these studies, i.e., VBM, which may not be optimal in accounting for the shape and size of subcortical structures (Zatorre et al. 2012). To overcome this, Burgaleta and colleagues used the shape analysis technique FIRST (Patenaude et al. 2011) which can account better for regional variations in the shape of subcortical structures. VBM performs tissue segmentation based on locally averaged segmentation of the grey matter, and usually requires arbitrary smoothing, and is, therefore, more sensitive to errors in tissue classification. On the other hand, the vertex analysis employed by FIRST is based on pre-determined shape and appearance models for each of the available subcortical structures, meaning that the structure boundaries are determined based solely on their geometry and location, and without the need of smoothing. This makes FIRST a method that is potentially more sensitive to the detection of subcortical boundaries, as well as changes in these boundaries. Burgaleta et al. compared simultaneous Catalan–Spanish bilinguals to Spanish monolinguals, and uncovered a range of significant between-groups effects: more specifically, compared with monolinguals, bilinguals demonstrated bilateral expansion of the putamen and the thalamus, as well as expansion of the right caudate nucleus (RCN) and the left globus pallidus. In interpreting their findings, Burgaleta and colleagues suggested that concurrent usage of two languages from an early age affects subcortical morphology. Moreover, they suggested that the putaminal effects replicate those reported by Abutalebi et al., and they attributed the bilaterality of their pattern to the different technique, sample size and AoA of the L2. The rest of the effects have not been reported in the structural literature before. Burgaleta et al. attributed the thalamic effects to increased need for speech monitoring in bilinguals, but recognised that the effects were widespread in several thalamic subnuclei, and, therefore, in need of further elaboration. The thalamus has been heavily implicated in cognitive control, including, but not limited to, control of language selection in bilinguals. For example, Abutalebi and Green (2016) suggested that, because of its extensive connections to the left inferior frontal gyrus and the basal ganglia (Ford et al. 2013), the thalamus is crucial in selecting among competing lexical and semantic representations during language production in bilinguals. Therefore, thalamic increases in simultaneous bilinguals might also reflect the lifelong necessity for language selection. Similarly, Burgaleta et al. attributed the effects in the RCN to its previously documented role in speech production (e.g., Grogan et al. 2009), and the pallidal effects to the reported importance of the globus pallidus in verbal fluency. Regarding the latter, Whelan et al. (2004) showed that patients with pallidotomy exhibit severe fluency deficits (see also York et al. 2003). However, the globus pallidus is rarely reported in the bilingual literature; for example, Stein et al. (2009) reported increased bilateral pallidal activation in bilinguals reading words in a third unknown language, but not when reading words in their L2, potentially suggesting a special role in the acquisition of a new language. In addition, Liu et al. (2010) reported increased bilateral pallidal activation in bilinguals for naming pictures in L2 (English) vs. in L1 (Chinese), suggesting that the globus pallidus is part of a wider network that monitors language control; moreover, they reported activation of the right globus pallidus for naming pictures in L1 vs. in L2, suggesting that the activation of this structure is related to the phonological and articulatory properties of a language. Summarising these effects, it appears that simultaneous bilingualism affects an extensive network of subcortical structures directly related to different stages of speech production, from articulation to speech monitoring and language selection.

If the reported effects on simultaneous bilinguals are a consequence of lifelong usage of two languages, it remains to be shown whether, and under which circumstances, similar effects would be observed in late sequential bilinguals, i.e., people that learnt their L2 at later age than their L1, usually during adolescence (Pliatsikas and Marinis 2013a). The absence of subcortical effects in the VBM literature may be related to the fact that most of the studies tested late learners of an L2, suggesting that, indeed, it is lifelong bilingualism that brings about subcortical effects. The only exception to this was Abutalebi et al. (2013), who attributed their structural findings in the late multilinguals to the *experience* of handling multiple languages. It is possible, indeed, that any effects reported in the early lifelong bilinguals are not necessarily due to simultaneous acquisition of two languages, but to the active and continuous usage of these two languages, or otherwise their *immersion* in a bilingual environment (Pliatsikas and Chondrogianni 2015). In a recent study, Pliatsikas et al. (2015) analysed the white matter structure of highly immersed young late bilinguals (mean L2 AoA: 10.15 years, mean L2 immersion: 91 months), with *immersion* defined as the amount of time they spent in a country (UK) where their L2 (English) was the official language. Pliatsikas and colleagues reported increased myelination for bilinguals, compared with age- and education-matched monolinguals, in a number of white matter tracts related to language processing. Importantly, their pattern of results closely resembled that of a previous study which tested elderly lifelong bilinguals with the same technique (Luk et al. 2011), suggesting that structural effects in the brain can be observed solely as a result of immersive bilingualism. Based on that finding, this study investigated whether immersive bilingualism also has an effect on the shape of subcortical structures. Using the protocol presented in Burgaleta et al. (2016), we compared the subcortical structure of two groups of sequential bilinguals with different amounts of L2 immersion, against monolingual controls: (a) the groups from Pliatsikas et al. (2015), including bilingual participants of high linguistic immersion in the UK, and (b) the groups from Pliatsikas et al. (2014a, b), including bilinguals of comparable L2 proficiency and AoA but with limited L2 immersion. If the previously reported subcortical effects are due to continuous usage of two languages, rather than their simultaneous acquisition, we would expect them to be replicated in our group of highly immersed sequential bilinguals too, but not in the group with limited immersion. This will confirm the hypothesis that structural changes in the brain are the result of increased processing demands for bilinguals, which are related to the amount of immersion in a bilingual environment.

## Experiment 1

### Methods

#### Participants

The group of participants from Pliatsikas et al. (2015) took part in this experiment. This included 20 L2 speakers of English with various L1 backgrounds (mean age 31.85, SD 8.06), which had lived in the UK for an average of 91 months (SD 84) at the time of testing (range 13–374 months), and had acquired English at a mean age of 10.15 years (SD 4.17) (sequential learners, Mohades et al. 2012). The proficiency of the participants was assessed with the Quick Placement Tests (QPT) (Geranpayeh 2003), in which they scored a mean 82.3 % (SD 12.55). Bivariate correlations were run for the three demographic factors (immersion, AoA, and proficiency) to assess whether they were independent from each other. There was no significant correlation between immersion and AoA [*r*(19) = −0.248, *p* = 0.291] and between proficiency and AoA [*r*(19) = −0.103, *p* = 0.664], and a significant positive correlation between immersion and proficiency [*r*(19) = 0.471, *p* = 0.036].

The bilingual participants were compared with 25 English native speaker controls (mean age 28.16; SD 5.33) who did not report speaking an L2. More details about the demographics of both groups can be found in Pliatsikas et al. (2015). This research was approved by the University of Reading Research Ethics Committee. Informed consent was obtained from all individual participants included in the study.

#### Data acquisition

A 3.0-Tesla Siemens MAGNETOM Trio MRI scanner with Syngo software and 32-channel Head Matrix coil was used. We acquired a T1-weighted MPRAGE (Magnetization Prepared Rapid Gradient Echo) brain scan from each participant (192 sagittal slices, 1 mm slice thickness, in-plane resolution 250 × 250, acquisition matrix of 246 × 256 mm, echo time = 3.02 ms, repetition time = 2020 ms., inversion time = 900 ms., flip angle = 9°). The scan lasted 10 min.

#### Data analysis

The images were preprocessed with FSL (Jenkinson et al. 2012): they were reoriented to MNI orientation, automatically cropped, bias-field corrected and non-linearly registered to the MNI space. Following that, subcortical structures were segmented with FIRST (Patenaude et al. 2011), an analysis tool implemented in FSL. Based on the effects reported in Burgaleta et al. (2016), we automatically segmented the thalamus, globus pallidus, putamen, and the caudate nucleus. Quality control of the segmented images was performed by an experienced researcher. No images were discarded as a result of it. The structures of interest were subsequently submitted to a vertex analysis, as implemented in FIRST. Following a standard procedure, each structure of interest underwent 6 degrees of freedom (three translations, three rotations) rigid body registration to sample-specific average surface that was in native space, i.e., not registered in a brain template in standard space. This way we ensured that we accounted for differences in orientation and location of the subcortical structures, while at the same time, preserving differences in shape and size could have been eliminated if the images were registered in standard space. Subsequently, the vertex coordinates for each structure of interest and participant were projected to the average coordinates of their group (monolinguals and bilinguals, respectively). For each participant, this created maps signifying vertex displacement that is perpendicular to the average surface, with positive values denoting displacement outside the surface and negative values denoting displacement inside the surface. These values were later analysed with a between-groups analysis with permutation-based non-parametric testing with Randomise (Winkler et al. 2014), corrected for multiple comparisons with Threshold-Free Cluster Enhancement (TFCE) (Smith and Nichols 2009). Two contrasts were examined: Bilinguals > Monolinguals and Monolinguals > Bilinguals (testing for subcortical expansions and contractions, respectively), and age and sex were included in the model as covariates of no interest. This analysis produced statistical images of the significant between-groups differences, which were thresholded at *p* < 0.05.

#### Correlations with immersion, proficiency, and age of acquisition

To determine whether any shape changes in the subcortical structures of interest were related to the above factors, the vertex analysis was re-run on the structures of interest and on the bilingual speakers only, with L2 immersion (in months), proficiency (QPT score), and AoA (in years) added as a predictors and age and sex added as covariates of no interest in separate analyses.

### Results

**Fig. 1:**
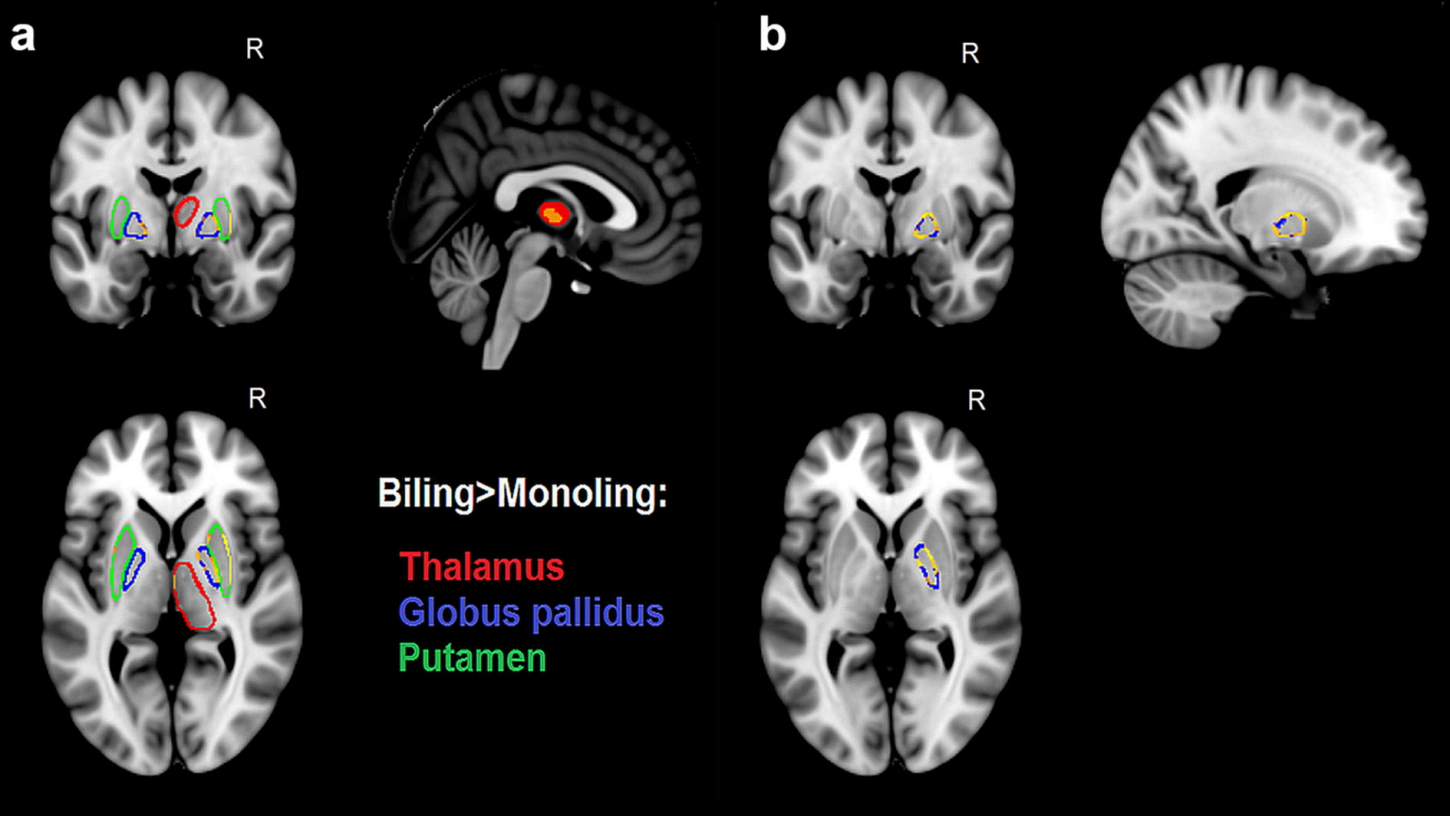
Effects of immersive bilingualism on the shape of subcortical structures. **a** Shows the significant differences between bilinguals and monolinguals, expressed as surface expansions for bilinguals in the bilateral globus pallidus (*blue*), bilateral putamen (*green*) and right thalamus (*red*). **b** Shows the portions of the right globus pallidus, where linguistic immersion emerged as a significant predictor of surface expansion. All effects are corrected for multiple comparisons with TFCE (*p* < 0.05) and illustrated in *yellow*

**Fig. 2:**
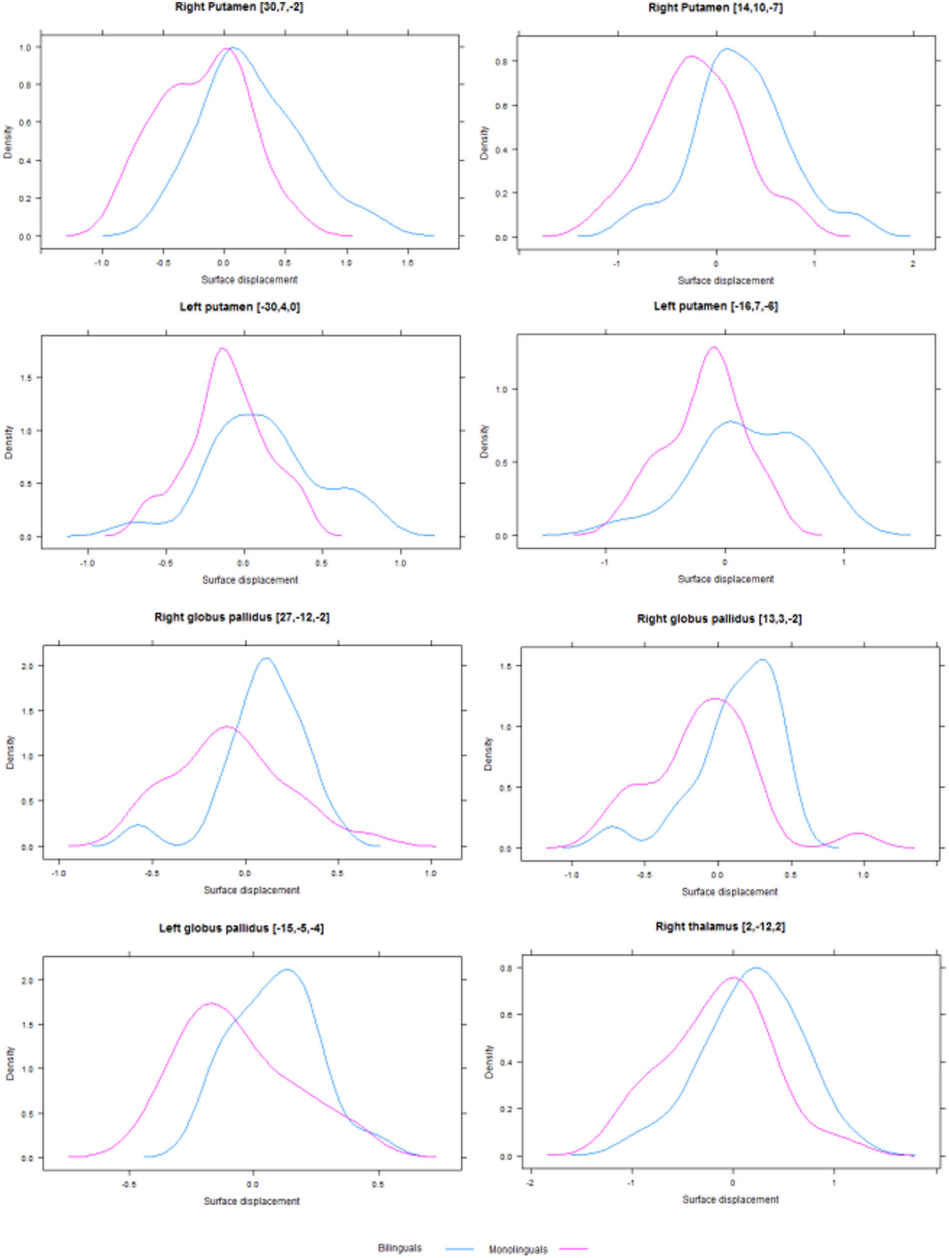
Density plots depicting surface displacements for both groups at the peak vertices in each affected structure. *0* represents no orthogonal displacement from the average surface across all participants

Our between-groups comparison revealed significant expansions in several subcortical structures for bilinguals compared with monolinguals, which are detailed below, along with the coordinates of the relevant peaks in standard space. First, we observed bilateral expansion of the putamen, with the right structure demonstrating larger effects in the central-posterior lateral surface (30, 7, −2) and some expansions in the central medial surface (14, 10, −7), whereas in the left structure, there were smaller expansions in anterior portions of both lateral (−30, 4, 0) and medial (−16, 7, −6) surfaces. Similarly, for the globus pallidus, we observed significant expansions in the posterior lateral (27, −12, −2) and anterior medial (13, 3, −2) surfaces of the right structure, and the left structure demonstrated expansions in a smaller anterocentral portion of the medial surface (−15, −5, −4). Finally, we found a significant expansion of medial surface of the right thalamus (2, −12, 2). Figure 1a illustrates these effects. No significant expansions were observed for the caudate nucleus, as well as no significant contractions for any of the structures on interest. Figure 2 displays density plots of surface displacement for bilinguals and monolinguals at the reported peaks for each affected structure.

Furthermore, our correlation with immersion on the bilingual data revealed that the time spent in the UK was a significant predictor for the expansion of the right globus pallidus. As illustrated in Fig. 1b, this appears to be a global effect on the structure, as immersion seems to predict the expansion of almost its entire surface. Similar effects only approached significance for the left globus pallidus (*p* = 0.087). To tease apart the effects of immersion from any potential effects of proficiency and AoA, we reran the immersion analysis with both factors added as covariates of no interest in separate models. The same pattern of results emerged from both analyses, further confirming that the observed effects can be attributed to linguistic immersion. However, the separate analyses with proficiency and AoA produced no significant effects.

## Experiment 2

The results of Experiment 1 revealed a pattern of significant surface changes in most subcortical structures of interest, and this pattern closely resembled the results presented by Burgaleta et al. (2016) in simultaneous bilinguals. Since our group in Experiment 1 consisted of highly proficient and highly immersed bilinguals, it can be concluded that the effects reported by Burgaleta and colleagues are not necessarily dependent on the early simultaneous acquisition of two languages or even lifelong bilingualism, but are a result of continuous active usage of two languages, and, therefore, achievable by highly immersed sequential bilinguals too. This suggestion is further supported by the finding that the amount of L2 immersion was a significant predictor of the pallidal changes. However, there is always the possibility that the observed effects are a direct consequence of the high L2 proficiency of the participants, rather than their linguistic experience per se. To further investigate this, we examined another group of highly proficient bilinguals but with limited immersion in an L2 speaking environment, which we compared with age-matched monolinguals. The absence of a similar pattern of effects in this bilingual group would further strengthen the hypothesis that the reported effects in Experiment 1, as well as in Burgaleta et al. (2016), can be attributed to active usage of two languages in an immersive environment for an extended amount of years.

### Methods

#### Participants

The participants from Pliatsikas et al. (2014a, b) took part in this experiment. This included a group of 17 Greek L2 learners of English (mean age 27.5 years, SD 5.55), who had learnt English at a mean age of 7.7 years (SD 2.2) (sequential bilinguals) and had lived in the UK for 3.97 years on average (SD 3.5, median 2.5, range 1–13)^1^. This group was also tested with the QPT (Geranpayeh 2003) and scored 82.4 % (SD 10). Therefore, the bilingual group in Experiment 2 had the same level of proficiency and comparable AoA with the group from Experiment 1, but approximately half the amount of L2 immersion. Similar to the result for the bilingual group in Experiment 1, bivariate correlations revealed that AoA did not correlate with immersion [*r*(16) = −0.045, *p* = 0.863] or with proficiency [*r*(16) = −0.033, *p* = 0.899], but immersion correlated positively with proficiency [*r*(16) = 0.602, *p* = 0.011]. The bilingual participants were compared to a group of 22 monolingual native speakers of English (mean age 24.5, SD 3.9). This research was approved by the University of Reading Research Ethics Committee. Informed consent was obtained from all individual participants included in the study.

#### Data acquisition

Whole-brain T1-weighted MPRAGE images were acquired with a 3.0-T Siemens MAGNETOM Trio MRI scanner with Syngo software and a 12-channel head matrix coil. These were high-resolution gradient-echo 3D anatomical images collected with 176 × 1 mm slices (TE, 2.52 ms.; TR, 2020 ms.; TI, 1100 ms.; FOV, 250 × 250 mm^2^). The scan lasted 5 min.

#### Data analysis

The same analysis protocol as in Experiment 1 was used. This included the separate analyses for the bilingual group, where immersion (in years), proficiency (QPT score), and AoA (in years) were added as predictors.

### Results

**Fig. 3:**
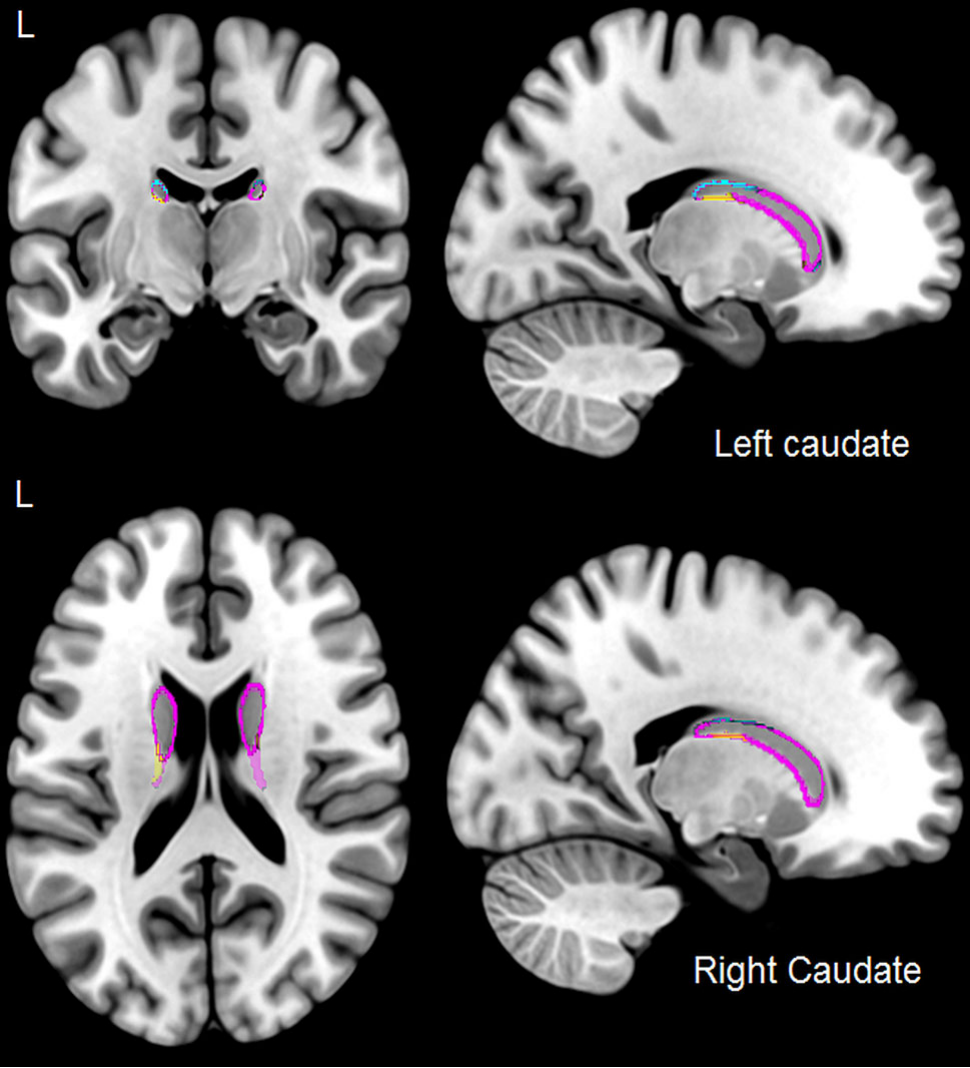
Effects of bilingualism on the bilateral caudate nucleus (*purple*) in the group with limited naturalistic immersion. Expansions are illustrated in *yellow*, and contractions in *blue*

**Fig. 4:**
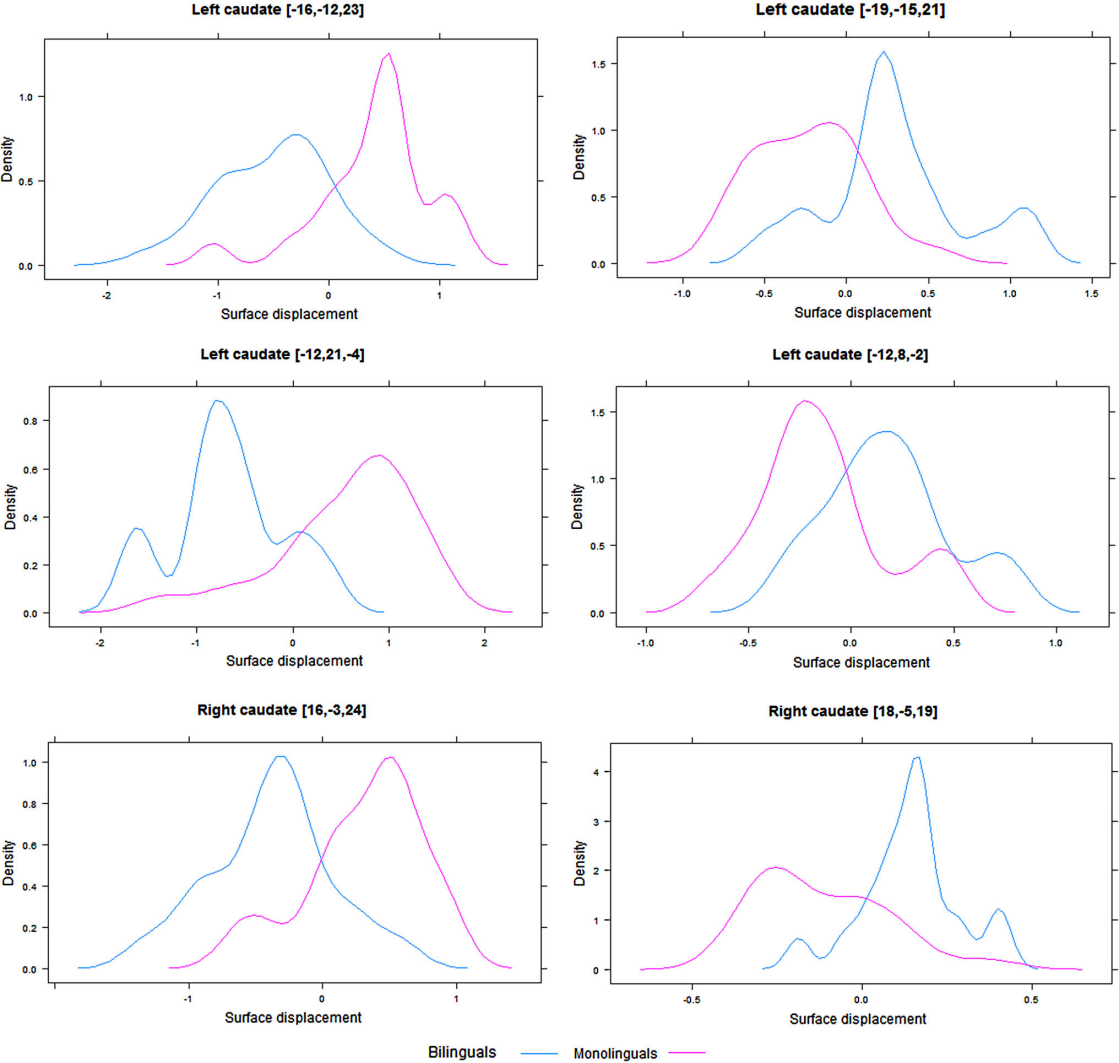
Density plots depicting surface displacements for both groups at the peak vertices in bilateral caudate nucleus. *0* represents no orthogonal displacement from the average surface across all participants

Our subcortical analysis revealed a significant expansion for the bilingual group at the lateral posterior inferior surface of the right caudate nucleus, which was accompanied by a significant contraction at the posterior superior surface, signifying overall reshaping of the structure. A similar pattern emerged in the left caudate nucleus: contraction of the posterior superior surface and expansion of the posterior inferior surface for bilinguals. A significant contraction on an anterior portion of the left structure was also observed, accompanied by an expansion of a more posterior portion of it, which only emerged in the uncorrected data. The significant effects in the bilateral caudate nucleus are illustrated in Fig. 3. Figure 4 displays density plots for these effects. Similar to the caudate nucleus, significant contractions were observed in bilateral thalamus and putamen. Table 1 illustrates these effects. The immersion, proficiency, and AoA analyses for the bilingual group produced no significant results.

**Table 1:** Peak coordinates of surface displacements in Experiment 2

Structure	Hemisphere	Monolinguals > Bilinguals			Bilinguals > Monolinguals		
		*x*	*y*	*z*	*x*	*y*	*z*
Caudate	L	−16	−12	23	−19	−15	21
		−12	21	−4	−12	8	−2*
	R	16	−3	24	18	−5	19
Thalamus	L	−8	−31	7			
		−6	−3	−1			
	R	13	−32	8			
		13	−6	3			
Putamen	L	−16	9	−11	−26	−11	1*
		−28	−17	8			
	R	29	−12	10			
		18	15	−10			

* Significant in the uncorrected data only (*p* < 0.01)

## Discussion

This study aimed to investigate the effects of immersive sequential bilingualism on subcortical brain structures. By utilising an analytical technique that is relatively new and under-used in the relevant literature, in Experiment 1, we set out to find whether the subcortical effects that have been reported in simultaneous bilinguals would be replicated in a group of highly immersed sequential bilinguals, and whether bilingual immersion is a significant predictor of these effects. In Experiment 2, we subsequently looked at a group of bilinguals with comparable L2 proficiency and AoA, but limited L2 immersion, to see whether a similar pattern of effects would emerge. Several significant effects emerged from our analysis on the immersed group, whereas the non-immersed group revealed very limited structural changes compared with the monolingual controls. The following paragraphs discuss the observed effects in relation to the role of the affected subcortical structures in language processing.

The first major effect of bilingualism observed in Experiment 1 is the bilateral expansion of the putamen, which was more widespread in the right structure. These effects essentially replicate the findings by Burgaleta et al. (2016), who nevertheless reported larger expansions at the left putamen. The putamen has long been considered an important structure in bilingual speech production, both in the early (Abutalebi et al. 2013) and late bilinguals. With respect to the latter, functional studies report increased unilateral and/or bilateral activity in the putamen in L2 tasks involving word and sentence reading (Dodel et al. 2005; Golestani et al. 2006), picture naming (Abutalebi et al. 2008; Liu et al. 2010), and translation between languages (Price et al. 1999). Since the putamen has been considered an important structure for the monitoring of articulation (Simmonds et al. 2011) and phonological errors (Tettamanti et al. 2005), the significant expansion observed in our study might reflect the increased articulation and language switching needs of highly immersed late bilinguals.

A small effect of bilingualism was also observed in the medial right thalamus of the highly immersed group. According to Llano (2013), the right thalamus is involved in a range of tasks tapping on fluency and word production. Similar to the effects for the putamen, this effect might further highlight the increased language control needs of sequential bilinguals, denoting an important thalamo-striatal network that monitors articulation and speech production on bilinguals, including selection among lexical and semantic alternatives (Abutalebi and Green 2016).

The other important finding of this study considers the significant bilateral expansion of the globus pallidus in our immersed bilinguals. The relevance of this structure in language production has been highlighted in various theoretical models, including suggestions for its role in semantic monitoring during speech production (Crosson et al. 2003). However, pallidal activity features less often on studies tapping on bilingual processing. For example, bilateral activity has been reported for L2 picture naming (Liu et al. 2010) and word reading (Stein et al. 2009), but the globus pallidus is notably absent from theoretical models considering the role of the basal ganglia in bilingual speech production and control (Green and Abutalebi 2013; Abutalebi and Green 2016). The significant effects of bilingualism on the globus pallidus reported both here and in Burgaleta et al. (2016), combined with the effects on the surrounding structures, further highlight the role of this structure in bilingual phonological processing. This is further supported by our finding that the changes in shape of the right globus pallidus positively correlate with the amount of L2 immersion of our bilinguals (the same effect did not reach significance in the left globus pallidus). If the globus pallidus does have a role in language production in bilinguals, this finding might indicate increased recruitment of this structure as a result of linguistic immersion. This, in turn, might reflect the progressive acquisition of the phonological and articulatory systems of the L2 as a function of linguistic experience and usage, an effect that has already been reported behaviourally (Flege and Liu 2001; Flege 2009).

The final structure of interest, namely the caudate nucleus, was significantly reshaped bilaterally in the less immersed group only (Experiment 2), but there were no effects in the immersed group (Experiment 1). This difference is of particular importance, as the caudate nucleus is frequently included in the networks that underlie language control, along with the putamen (Green and Abutalebi 2013; Abutalebi and Green 2016). More specifically, the LCN has been suggested to underlie language selection in bilinguals, and reduce interference from the non-target language (Green and Abutalebi 2013). The absence of any effects for our immersed group and the simultaneous bilinguals in Burgaleta et al., combined with our findings from the less immersed group, suggests that this region may be more relevant to the processing of a less proficient or exercised L2, or utilised during the initial stages of L2 acquisition (Abutalebi and Green 2007). This explanation also accounts for the previous findings on LCN in bilinguals with limited immersion (e.g., Abutalebi et al. 2008) and suggests that active immersive bilingualism results in more efficient language switching and/or suppression of the non-target language, which eventually removes the need for the observed changes on the LCN. The absence of an effect in the RCN for the immersed group is more difficult to explain, especially since significant reshaping is reported in both our less immersed group and the simultaneous bilinguals in Burgaleta et al. The RCN is less frequently reported in the bilingual literature, and it has been suggested to underlie native-like L2 grammatical processing (Pliatsikas et al. 2014b), to share some of the switching functions of the LCN (Wang et al. 2007; Luk et al. 2012), as well as to underlie phonemic fluency (Grogan et al. 2009), including a positive correlation between its size and the bilinguals’ performance in fluency tasks. In any case, the effects on the RCN on both simultaneous bilinguals and less immersed bilinguals suggest that its role in bilingual processing might be independent of L2 immersion or AoA. The absence of RCN effects in our immersed group might possibly be due to the variety of L1s that the immersed group had, in contrast to the uniform L1s of the other two groups. Similarly, the significant contractions in the less immersed group are difficult to account for, especially when they were not accompanied by significant expansions on the same structures, which would signify global reshaping. It is worth noting though that there are only small discrepancies between Experiment 1 and Burgaleta et al., but a very different pattern of results in Experiment 2. Taken together, these results suggest that active language use is an important predictor of structural changes in the brain that are induced by bilingualism.

An important limitation in our study is the absence of behavioural measures that could be used to determine whether the observed effects are indeed a result of the phonological acquisition of the L2 or of bilingual language control, as the existing theoretical models largely argue. Other explanations cannot be readily dismissed: for example, the basal ganglia have been implicated in the processing of grammar (Ullman 2004), so our findings may reflect the progressive acquisition of grammatical features in L2 as an effect of immersion (see Pliatsikas and Marinis 2013b, for related behavioural evidence), or even continuous handling and control of two grammatical systems. However, the available neuroimaging literature hardly, if at all, implicates the basal ganglia and the thalamus in L2 grammatical processing (for recent reviews see Roberts et al. 2016, in press; Roncaglia-Denissen and Kotz 2016). Similarly, the basal ganglia have been implicated to cognitive processing in domains beyond language, notably in executive functions (Graybiel 2000), a domain in which bilinguals are reported to have certain advantages over monolinguals (Bialystok 2016). Our behavioural assessment, the QPT, cannot safely differentiate between the candidate explanations, as it simply is an offline language aptitude test which is not designed to test linguistic or cognitive theories. Future studies focusing on the structure of the bilingual brain should be accompanied by behavioural measures that are related to the proposed functions of the areas of interest. We also recognise that the acquisition of linguistic skills in different domains (e.g., syntax, phonology) might be affected differently by the L2 AoA. Therefore, any suggestions related to the effects of AoA on the structure of the brain should be taken with caution, especially when they are linked to a specific linguistic skill in the absence of appropriate behavioural measures, but also in groups with limited AoA range, which this study provided by design.

To conclude, this study reports significant effects of immersive sequential bilingualism on the shape of the basal ganglia and the thalamus. Importantly, our participants were highly proficient and highly immersed learners of L2 English, while the pattern of effects resembles the previously reported pattern for lifelong simultaneous bilinguals. Another finding was that the amount of time spent in an immersive bilingual environment correlated positively with some of the structural effects. None of these effects emerged in a group of bilinguals with limited immersion and comparable L2 proficiency and AoA. Taken together, these effects suggest that second language acquisition, as well as its structural correlates in the brain, is a dynamic procedure that is highly related to the amount of immersion in a bilingual environment. In other words, structural effects pertinent to simultaneous bilinguals, as well as the cognitive effects they may convey, are applicable to the late simultaneous bilinguals, as long as language acquisition and use is active.

## Footnotes

1 Note that UK residence was reported in years in Pliatsikas et al. (2014a, b), and in months in Pliatsikas et al. (2015). We chose not to convert the former into months, as it would give us an inaccurate figure- for example, we can anecdotally report that several participants in the 2014 study had immersion of a few months, which they rounded up to a year in order to conform to the question in the relevant questionnaire.
